# Proteomic Profiling Reveals TPR and FGA as Predictive Serum Biomarkers of Relapse to First- and Second-Generation EGFR-TKIs in Advanced Lung Adenocarcinoma

**DOI:** 10.3390/biomedicines13071608

**Published:** 2025-06-30

**Authors:** Pritsana Raungrut, Wararat Chiangjong, Thipphanet Masjon, Saowanee Maungchanburi, Thidarat Ruklert, Narongwit Nakwan

**Affiliations:** 1 Division of Biomedical Sciences and Biomedical Engineering, Faculty of Medicine, Prince of Songkla University, Hat Yai 90110, Songkhla, Thailand; thippanet9213@gmail.com (T.M.); msaowane@medicine.psu.ac.th (S.M.); 2 Pediatric Translational Research Unit, Department of Pediatrics, Faculty of Medicine Ramathibodi Hospital, Mahidol University, Bangkok 10400, Thailand; wararat_01@yahoo.com; 3 Division of Pulmonology, Department of Medicine, Hat Yai Medical Education Center, Hat Yai Hospital, Hat Yai 90112, Songkhla, Thailand; thidaratruklerd@gmail.com (T.R.); naronak@hotmail.com (N.N.)

**Keywords:** TPR, FGA, serum biomarkers, relapse, EGFR-TKIs, proteomic, lung adenocarcinoma

## Abstract

**Background**: Epidermal growth factor receptor tyrosine kinase inhibitors (EGFR-TKIs) significantly enhance the median survival of patients with lung adenocarcinoma (ADC) that harbor EGFR-sensitive mutations. However, most patients inevitably experience tumor relapse owing to drug resistance. We aimed to identify potential serum biomarkers for predicting post-EGFR-TKI treatment relapse in patients with advanced-stage lung ADC. **Methods**: Among 27 patients, including 6 and 21 with early and late relapse, respectively, differentially expressed proteins between patients with early and late relapses were identified using liquid chromatography and tandem mass spectrometry and subsequently validated using Western blotting. Predictive ability was assessed using the receiver operating characteristic curve and area under the curve (AUC) analysis. The association between the clinical variables and treatment response was evaluated using the chi-square test. **Results**: The serum expression levels of the translocated promoter region (TPR), junction plakoglobin (JUP), and fibrinogen alpha chain (FGA) were significantly higher in patients with late rather than early relapse. The findings indicated that TPR and FGA exhibited good diagnostic performance, with AUCs of 0.946 (*p* = 0.002; 95% confidence interval [CI], 0.84–1.05) and 0.809 (*p* = 0.034; 95% CI, 0.65–0.97), respectively. **Conclusions**: Our results suggest that the TPR and FGA levels are potential predictors of post-EGFR-TKI treatment relapse.

## 1. Introduction

Non-small-cell lung cancer (NSCLC) constitutes approximately 85% of all lung cancer cases [1], with adenocarcinoma (ADC) being the predominant histological subtype, which accounts for 40% of all NSCLC cases [2]. Epidermal growth factor receptor tyrosine kinase inhibitors (EGFR-TKIs) include the first generation (e.g., gefitinib, erlotinib, and icotinib) and second generation (e.g., afatinib and dacomitinib). These have been approved worldwide as first-line treatments for lung ADC with epidermal growth factor receptor (EGFR) mutations, which occur in 40–60% and 10% of Asian and Western populations, respectively [3]. Compared with platinum-based chemotherapy, EGFR-TKIs, with their response rate of 60–80%, significantly prolong the median survival of patients with advanced lung ADC [4,5]. However, acquired resistance to EGFR-TKIs, which is the emergence of drug resistance after an initial response to treatment over diverse intervals [6], occurs in nearly all patients after treatment. Several studies have indicated a notable trend of early tumor relapse, within 3–6 months, with EGFR-TKI treatment. Treatment with erlotinib, gefitinib, or afatinib was associated with a progression-free survival (PFS) of approximately 16.7%, 13.1%, 18.0%, 16.3–18.8%, and 14.3–16.8% in Japanese [7], Indian [8], Chinese [9], Korean [10], and Taiwanese [11] patients, respectively, whereas Polish patients had a longer PFS of 21.8–33.9% [12]. These lines of evidence suggest the possibility of early relapse following an initial response to EGFR-TKIs.

Numerous studies have shown that genetic mutations, including EGFR, influence the mechanism of acquired resistance. The presence and persistence of the T790M mutation in EGFR and concurrent genomic alterations in other genes such as TP53 and MET exemplify this phenomenon [3,7,10,13]. However, the occurrence of DNA mutations does not inherently imply that the expression of downstream proteins that are responsible for inducing acquired resistance is affected. In addition, there are presently no proteins in the blood that are employed as biomarkers to distinguish the response status and relapse of EGFR-TKI treatments. In this study, based on a proteomic analysis of patients with lung ADC, we aimed to identify potential blood-based proteins that may predict relapse in patients with acquired resistance to EGFR-TKI treatment.

## 2. Materials and Methods

### 2.1. Participant Selection

Serum samples were collected from 27 patients with inoperable NSCLC who were diagnosed with lung ADC between 2022 and 2024. The study protocol was approved by the Human Research Ethics Committee of the Hat-Yai Hospital, Songkhla, Thailand. Written informed consent was obtained from all the participants. The inclusion criteria were (1) a recent diagnosis of pathologically confirmed Stage III or IV cancer, and (2) administration of first- or second-generation EGFR-TKIs as the first-line treatment. Patients with prior lung cancer treatment and those who were lost to follow-up or died during treatment were excluded. Computed tomography of the chest was performed before and after treatment every month to evaluate the treatment response.

### 2.2. Treatment Evaluation

The Response Evaluation Criteria in Solid Tumors (RECIST) was employed to assess the response of a tumor to treatment [14], which was categorized as follows: complete response (CR), which was the disappearance of all target lesions; partial response (PR), which involved a reduction of at least 30% from the total number of target lesions; stable disease (SD), which was the absence of lesion reduction or progression; and progressive disease (PD), which was the presence of new lesions or a progression of at least 20% among target lesions. Early relapses were defined as PD within 6 months, whereas late relapses were defined as PR, SD, or PD without disease recurrence for more than 6 months.

### 2.3. Serum Preparation

Blood samples (10 mL) were obtained at diagnosis, allowed to clot for 30 min at room temperature, and subsequently centrifuged at 3400× *g* for 10 min to collect the serum. The serum was filtered using a polyvinylidene difluoride syringe filter (pore size 0.22 mm; Merck Millipore, Darmstadt, Germany), and aliquots were stored at −80 °C until analysis.

### 2.4. Serum Depletion

Human serum albumin and major subclasses of gamma globulin were removed using the Pierce Albumin/IgG Removal Kit (Thermo Fisher Scientific, Waltham, MA, USA). The depletion column was created by adding 170 µL of the gel slurry into a spin column, which was subsequently centrifuged for 1 min at 10,000× *g* to remove the excess liquid. Next, 10 μL serum was diluted with binding/wash buffer to a final volume of 75 µL, and then applied to a gel bed column, incubated for 10 min at room temperature, and centrifuged at 10,000× *g* for 1 min. The flow-through was reapplied to the column. Following a second incubation and centrifugation, 75 µL binding/wash buffer was added to the same column and centrifuged to collect the wash. The flow-through and wash fractions were combined and classified as the unbound fractions. The bound fraction was eluted from the column using 1× sodium dodecyl sulfate sample buffer free of reducing agents. Both unbound and bound fractions were stored at −80 °C until further analysis.

### 2.5. In-Solution Digestion

After depleting the abundant protein in the serum, the Bradford assay (Bio-Rad Laboratories, Hercules, CA, USA) was used to quantify the total protein content. The 3 kDa cutoff spin filter was used to purify the serum protein amount (20 μg) of each sample. Briefly, the protein in the spin filter was subsequently reduced with 5 mM dithiothreitol in 8 M urea/50 mM Tris hydrochloride (Tris-HCl) (pH 8) at 37 °C for 1 h. The solution was concentrated by centrifugation at 12,000 rpm for 10 min; 250 μL 15 mM iodoacetamide in 8 M urea/50 mM Tris-HCl, pH 8 was added, and the filter was then incubated in the dark at room temperature for 1 h with agitation. The spin filter was centrifuged at 12,000 rpm for 10 min; then, we added 500 μL 50 mM ammonium bicarbonate. This step was repeated three times. Proteins were digested at 37 °C for 16 h with agitation using trypsin at a final ratio of 1:50 (*w*/*w*; Promega Corp., Madison, WI, USA), and the reaction was terminated by adding 10 μL 5% formic acid in 50% acetonitrile. The spin filter containing the peptides was then centrifuged at 14,000 rpm for 20 min. The peptides were desalted using C18 solid phase extraction disks (EMPORE™-3M, Saint Paul, MN, USA) and C18 beads. After purification, the peptides were dried using a vacuum centrifugal concentrator (LabConco, Kansas City, MO, USA) and resuspended in 0.1% formic acid to a final volume of 20 μL. The peptide solutions were then used for Sequential Window Acquisition of all Theoretical fragment ion spectra mass spectrometry (SWATH-MS) analysis.

### 2.6. SWATH-MS Analysis

Targeted label-free proteomic analysis was performed to explore the potential mechanisms of the samples. An amount of peptide sample corresponding to 2 μg of total protein was analyzed by an Eksigent nanoLC ultra nanoflow high-performance liquid chromatography coupled with a TripleTOF 6600+ mass spectrometer (Sciex, Toronto, Canada) in information-dependent acquisition (IDA) and data-independent acquisition (DIA) modes. The peptides were loaded onto a C18 column trap (Nano Trap RP-1, 3 μm 120 Å, 10 mm × 0.075 mm; Phenomenex, CA, USA) at a flow rate of 3 μL/min 0.1% formic acid for 10 min to desalt and concentrate the sample before separating with a C18 analytical column (bioZen Peptide Polar C18 nanocolumn, 75 μm × 15 cm, C18 particle sizes of 3 μm, 120 Å; Phenomenex, Torrance, CA, USA). The mass spectrometry (MS) conditions were as described by Khaosuwan et al. [15]. Peptide elution was performed at the flow rate of 300 nL/min with gradients of 3–30% acetonitrile/0.1% formic acid for 60 min, 30–40% acetonitrile/0.1% formic acid for 10 min, 40–80% acetonitrile/0.1% formic acid for 2 min, 80% acetonitrile/0.1% formic acid for 6 min, 80–3% acetonitrile/0.1% formic acid for 2 min, and then 3% acetonitrile/0.1% formic acid for 25 min. The eluate was ionized and sprayed into the mass spectrometer using OptiFlow Turbo V Source (Sciex). Ion source gas 1 (GS1), 2 (GS2), and curtain gas were set at 19, 0, and 25 vendor arbitrary units, respectively. The interface heater temperature and ion spray voltage were kept at 150 °C and 3.3 kV, respectively.

The MS was operated in positive ion mode and set for 3500 cycles for 105 min gradient elution. Each cycle performed 1 time of flight (TOF) scan (250 ms accumulation time, 350–1250 *m*/*z* window with a charge state of +2) followed by IDA of the most intense 100 ions, while the minimum MS signal was set to 150 counts. MS/MS scan was operated in a high-sensitivity mode with 50 ms accumulation time and 50 ppm mass tolerance. Former MS/MS candidate ions were excluded for a period of 12 s after their first occurrence to reduce the redundancy of identified peptides. DIA mode was performed in a range of 350 to 1500 *m*/*z* using a predefined mass window of 7 *m*/*z* with an overlap of 1 *m*/*z* for 157 transmissible windows. MS scan was set at 2044 cycles, where each cycle performed 1 TOF-MS scan type (50 ms accumulation time across 100–1500 precursor mass range) acquired in every cycle for a total cycle time of 3.08 s. MS spectra of 100–1500 *m*/*z* were collected with an accumulation time of 96 ms per SWATH window width. The resolutions for MS1 and SWATH-MS2 scans were 35,000 and 30,000, respectively. A rolling collision energy mode with a collision energy spread of 15 eV was applied. The IDA and DIA data (.wiff) were recorded by Analyst-TF v.1.8 software (Sciex).

A total of 24 wiff files of IDA experiments were combined and searched using Protein Pilot v.5.0.2.0 software (Sciex) against Swiss-Prot database (UniProtKB 2022_01) Homo sapiens (20,385 proteins in database) with the searching parameters as follows: alkylation on cysteine by iodoacetamide, trypsin enzymatic digestion, 1 missed cleavage allowed, monoisotopic mass, and 1% false discovery rate. The group file (Protein Pilot search result) was loaded into SWATH Acquisition MicroApp v.2.0.1.2133 in PeakView software v.2.2 (Sciex) to generate a spectral library. The maximum number of proteins was set as those identified at 1% global false discovery rate (FDR) from fit. Retention time alignment was performed by the high abundance of endogenous peptides covering the chromatographic range. SWATH data extraction of 24 DIA files (1 technical replication/sample) was performed by SWATH Acquisition MicroApp (Sciex) using the following parameters: 100 min extraction window, 20 peptides/protein, 6 transitions/peptide, excluding shared peptides, peptide confidence > 99%, FDR < 1%, and extracted ion chromatogram width of 50 ppm. SWATH extraction data, including the identities and quantities of peptides and proteins, was exported into an Excel file for statistical analysis using MarkerView version 1.3.1 (Sciex). Log2 fold-change (log2 FC) > 1.5 and *p* ≤ 0.05 were used as cutoffs for differentially expressed proteins (DEPs).

### 2.7. Bioinformatics Analysis

Data visualization, including cluster heatmaps, volcano plots, and multi-group bubble plots, was performed using the Science and Research online plot tool (SR plot) (https://www.bioinformatics.com.cn/en, accessed on 28 June 2025). Gene Ontology (GO) and Reactome Pathway Enrichment analyses of the DEPs were conducted using the Database for Annotation, Visualization, and Integrated Discovery (DAVID; https://david.ncifcrf.gov/tools.jsp, accessed on 28 June 2025). Gene Expression Profiling Interactive Analysis version 2 (GEPIA2; http://gepia2.cancer-pku.cn/#index, accessed on 28 June 2025) was used to evaluate gene expression levels between lung ADC and normal lung tissues as well as the overall survival (OS) and disease-free survival (DFS) of patients with lung ADC. The best cutoff value was auto-selected, and hazard ratios (HRs) were calculated using the Cox proportional hazards model. The Human Protein Atlas (HPA) database (http://www.proteinatlas.org/, accessed on 28 June 2025) was used to compare the protein expression of candidate genes in lung ADC specimens with those in normal lung specimens.

### 2.8. Serum Western Blotting

The expression levels of potential serum proteins were determined by Western blotting of individual samples using the method described by Raungrut et al. [16,17]. Serum was diluted with distilled water (1:5000), and protein concentrations were measured using the Bradford assay (Bio-Rad Laboratories). The serum protein (20 μg/well) was separated using 12% sodium dodecyl sulfate–polyacrylamide gel electrophoresis (SDS-PAGE) and subsequently transferred to a 0.22 μm nitrocellulose membrane (Bio-Rad Laboratories) at 4 °C overnight. Membranes were blocked with 3% bovine serum albumin for 90 min and further incubated overnight at 4 °C with primary antibodies diluted 1:5000 for rabbit polyclonal translocated promoter region (TPR) (Affinity Biosciences, Cincinnati, OH, USA), 1:10,000 for rabbit polyclonal fibrinogen alpha chain (FGA) (Santa Cruz Biotechnology, Inc., Dallas, TX, USA), and 1:500 for rabbit polyclonal junction plakoglobin (JUP) (Affinity Biosciences). The membranes were washed with Tris-buffered saline containing 0.1% Tween 20 and then incubated with horseradish peroxidase-conjugated anti-rabbit antibodies (Cell Signaling Technology, Danvers, MA, USA) at a dilution of 1:3000 at room temperature for 1 h. Bands on the membrane were identified using Clarity Western ECL substrate (Bio-Rad Laboratories). Abundant proteins retained on SDS-PAGE at a molecular weight of 37–50 kD were used as the loading control. The gel was fixed using 50% (*v*/*v*) ethanol (Merck Millipore) in water containing 10% (*v*/*v*) acetic acid (Merck Supelco, Darmstadt, Germany) for 1 h, and then washed with 50% (*v*/*v*) methanol (Merck Millipore) in water with 10% (*v*/*v*) acetic acid for 2 h. The gel was stained with 0.1% (*w*/*v*) Coomassie blue (Sigma-Aldrich, St. Louis, MO, USA) containing 20% (*v*/*v*) methanol and 10% (*v*/*v*) acetic acid until the development of a consistent blue color. The gel was treated with a destaining solution of 50% (*v*/*v*) methanol in water with 10% (*v*/*v*) acetic acid until the background staining diminished. All images were acquired using an ImageQuant LAS 4000 digital imaging system (GE Healthcare, Little Chalfont, UK). Band intensity was quantified using ImageJ version 1.52a. The relative expression of each protein was calculated by using the most abundant protein on the gel.

### 2.9. Statistical Analysis

Descriptive statistics for categorical variables were presented as percentages, whereas continuous variables were expressed as mean ± standard deviation. The associations among clinicopathological variables, protein expression, and relapse status were examined using the chi-square test. Using an optimal cutoff point that maximized the sum of sensitivity and specificity in the receiver operating characteristic (ROC) curve analysis, the expression levels of each protein were divided into low and high expression levels for further analysis. The corresponding areas under the curves (AUCs) were calculated using a logistic regression model. Statistical analyses were performed using GraphPad Prism 5.0 (GraphPad Software, Inc. Boston, MA, USA). An independent *t*-test was used for between-group comparison. Statistical significance was set at *p* < 0.05. 

## 3. Results

### 3.1. Clinicopathological Characteristics of Participants

Serum samples from 27 inoperable patients with ADC (21 male and 6 female) who were treated with EGFR-TKI-targeted therapy were analyzed. The median age was 60.0 years (range 39–84 years). All patients received first- or second-generation EGFR-TKI therapy, with 19 (74.1%), 5 (18.5%), and 2 (7.4%) receiving erlotinib, gefitinib, and afatinib, respectively. Most EGFR mutations were in exon 19 del (70.4%; 19/27 of all patients), followed by exon 21 L858R (25.9%; 7/27 of all patients). According to the RECIST criteria, 6 (22.2%) and 21 (77.8%) patients had early and late relapse, respectively (Table 1).

### 3.2. Differentially Expressed Serum Proteins Between Patients with Early and Late Relapse

**Figure 1:**
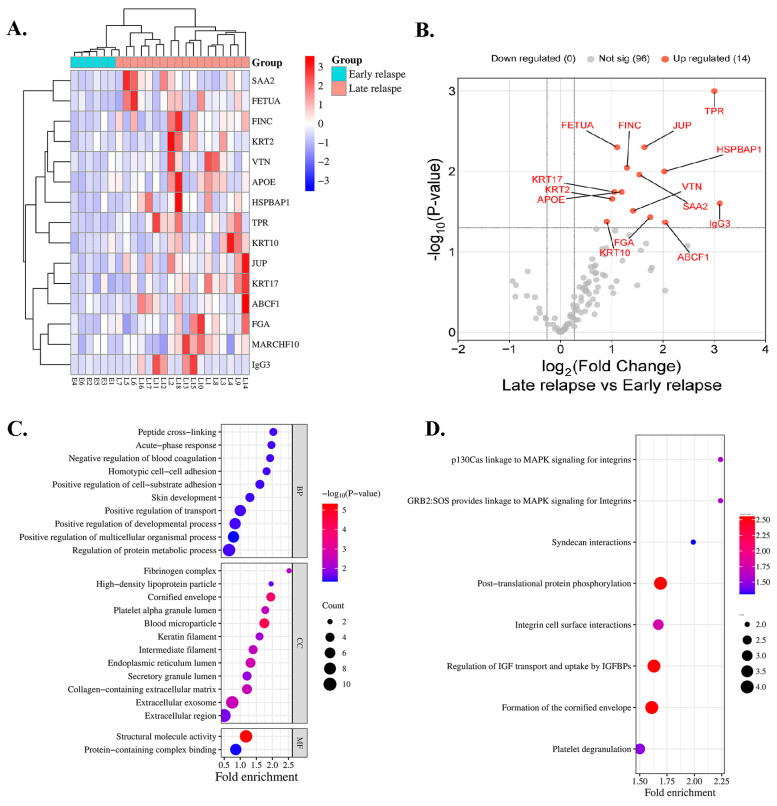
Protein expression profile of patients treated with EGFR-TKIs categorized by early and late relapse using proteomic analysis. (**A**) Heatmap of serum proteins exhibiting differential expression. Red indicates high expression, whereas blue indicates low expression. (**B**) Serum proteins that exhibit significant differential abundance are illustrated in the volcano plot. (**C**) GO classification and (**D**) reactome pathway enrichment analysis of differentially expressed serum proteins.

A total of 24 serum samples from 6 and 18 patients with early and late relapse, respectively, were included for proteomic profiling. In total, 111 proteins were identified in all cases. A heatmap indicated that 14 proteins exhibited differential expressions in the serum of patients with early relapse (E1–E6) compared with those with late relapse (L1–L18) of lung ADC, with a *p* < 0.05 (Figure 1A), and all proteins were upregulated (Figure 1B). Among the 14 analyzed proteins, 7 were classified as DEPs based on a log2 FC of ≥1.5 and an adjusted *p* < 0.05 (Table 2). Compared with patients with early relapse, the expression of TPR and immunoglobulin-heavy constant gamma 3 (IGHG3) was approximately 3.00-fold higher in patients with late relapse. Five proteins exhibited a fold-change > 1.5, including ATP-binding cassette sub-family F member 1 (ABCF1), HSPB1-associated protein 1 (HSPBAP1), FGA, JUP, and serum amyloid A-2 protein (SAA2) (Table 2).

### 3.3. Bioinformatics and Enrichment Analysis

Key biological processes (BPs), cellular components (CCs), and molecular functions (MFs) were identified using GO enrichment analysis. The most enriched BPs were involved in positive regulation of multicellular organismal processes. In the blood microparticles and cornified envelope, CCs were substantially enriched, whereas MFs highlighted structural molecular activity and protein-containing complex binding (Figure 1C). Reactome enrichment analysis demonstrated substantial enrichment in pathways that were associated with posttranslational protein phosphorylation, regulation of insulin-like growth factor (IGF) transport, uptake by insulin-like growth factor binding proteins (IGFBPs), and formation of the cornified envelope (Figure 1D).

### 3.4. Expression Profile of Differentially Expressed Serum Proteins in Lung ADC

**Figure 2:**
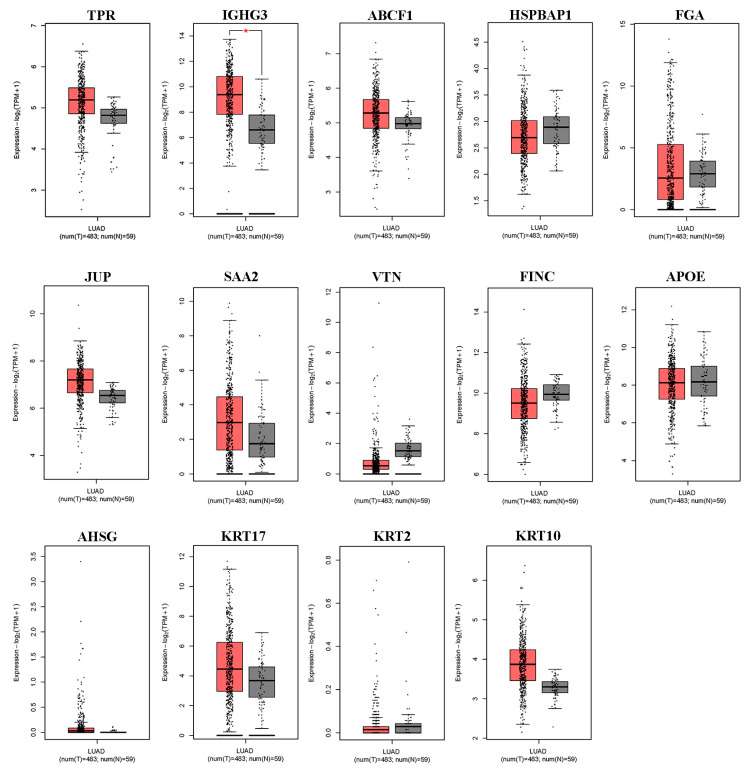
The expression levels of the 14 differentially expressed proteins in NSCLC tissues derived from the GEPIA2 database. Box plots illustrate the expression levels of TPR, IGHG3, ABCF1, HSPBAP1, FGA, JUP, SAA2, VTN, FINC, APOE, AHSG, KRT17, KRT2, and KRT10 in 483 lung adenocarcinoma (LUAD) tissues (T), represented in red, relative to the corresponding 53 normal lung tissues (N), represented in gray. * significant differences identified using the ANOVA test (*p* < 0.05).

We first used the online database GEPIA2 for cancer tissues and normal tissues from The Cancer Genome Atlas (TCGA) to verify the differential expression of DEPs in lung ADC. As shown in Figure 2, of the 14 differentially expressed serum proteins that were evaluated, only IGHG3 was significantly upregulated in lung ADC tissue. Other DEPs (TPR, ABCF1, HSPBAP1, FGA, JUP, SAA2, VTN, FINC, APOE, AHSG, KRT17, KRT2, and KRT10) showed no differential expression between lung ADC and normal tissues.

### 3.5. Prognostic Value and Expression Level of DEPs in Lung ADC Tissues

**Figure 3:**
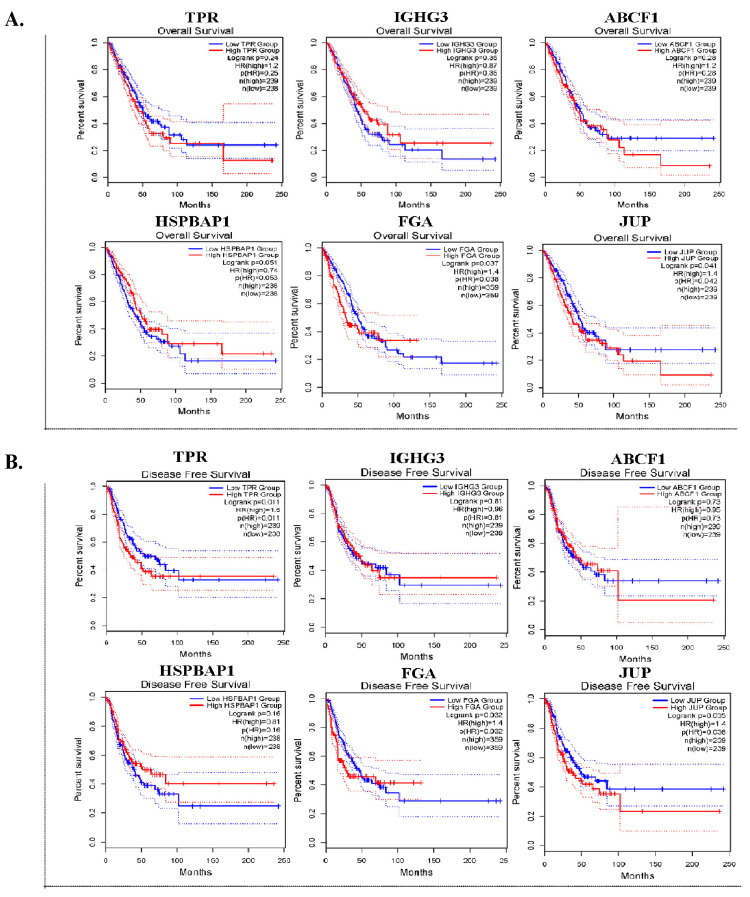
The prognostic significance of six DEPs in lung ADC tissues. Kaplan–Meier survival curves for overall survival (OS) (**A**) and disease-free survival (DFS) (**B**) of TPR, IGHG3, ABCF1, HSPBAP1, FGA, and JUP in patients with lung ADC.

Six DEPs with a log2 FC > 1.6 were selected for further validation. We conducted OS and DFS analyses by utilizing an online tool with data that were obtained from TCGA and GTXs via the GEPIA2 database. In the OS analysis, the expression of FGA (*p* = 0.037) and JUP (*p* = 0.041) was significantly associated with worse survival (Figure 3A). In DFS analysis, high TPR (*p* = 0.011), FGA (*p* = 0.032), and JUP (*p* = 0.035) expression levels were significantly associated with unfavorable DFS in patients with lung ADC (Figure 3B).

### 3.6. Expression of Selected DEPs in the HPA Online Tool

**Figure 4:**
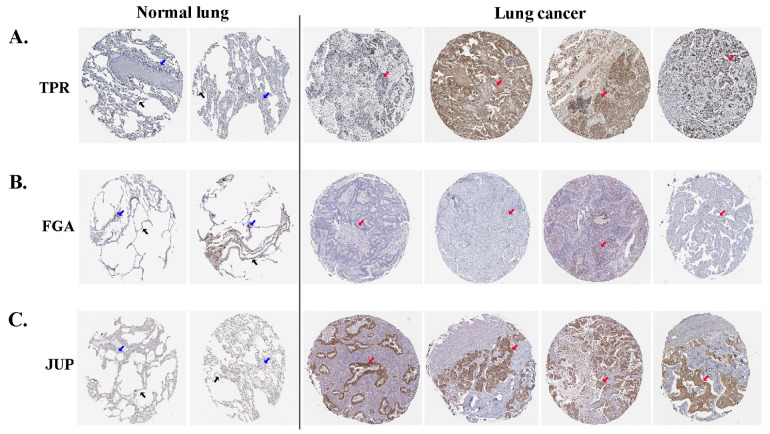
The analytical findings from the HPA database indicate the expression levels of TPR (**A**), FGA (**B**), and JUP (**C**) proteins in lung ADC and normal lung tissues. Alveolar cells are denoted by black arrows, macrophage cells by blue arrows, and tumor cells by red arrows.

Immunohistochemical images from the HPA were used to directly examine the expression of selected proteins between normal and lung ADC tissues. The HPA database revealed that TPR exhibited moderate expression in the nucleus of alveolar cells in normal lung tissues; however, in tumor cells of lung ADC, it was present in either the nucleus or cytoplasmic/membranous regions and displayed varying levels of expression from low to high (Figure 4A). FGA expression was negative in normal alveolar lung tissues, which is similar to that observed in most ADC lung tissues (Figure 4B). Negative JUP expression was absent in normal alveolar lung tissues, whereas medium to high expression was noted in the cytoplasmic and membranous regions of the lung ADC tissues (Figure 4C). These results indicated that the three differentially expressed proteins (TPR, FGA, and JUP) may be relevant in predicting the likely progression and outcome of the disease in patients with lung ADC.

### 3.7. Association of Treatment Response with Variables and Protein Expressions

**Figure 5:**
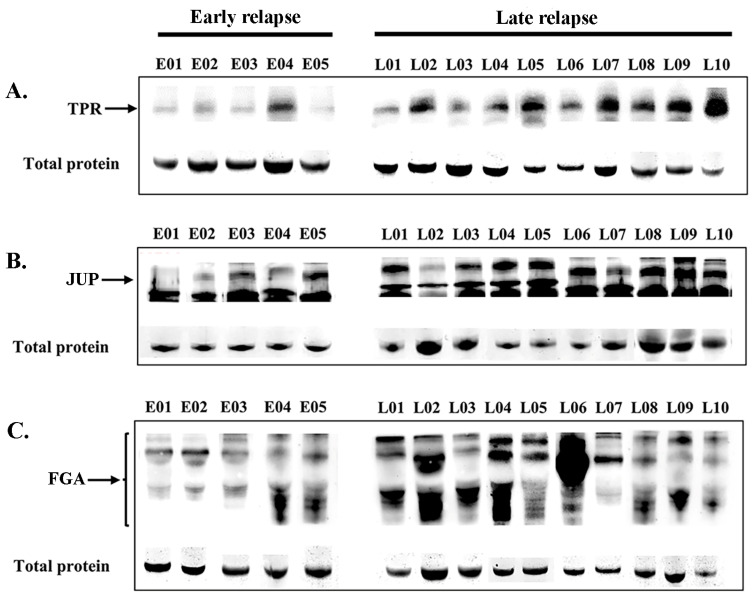
Serum expression levels (Western blotting) of TPR (**A**), JUP (**B**), and FGA (**C**) proteins in patients with early relapse compared with those with late relapse. The residual proteins on SDS-PAGE were used as an internal control.

Following online verification, three candidate DEPs were selected for validation in the sera of patients with lung ADC who received EGFR-TKI treatment. The expression levels were confirmed in the serum of 27 patients (early relapse, n = 6; late relapse, n = 21). Western blot analysis revealed the expression levels of TPR (267 kDa, Figure 5A), JUP (82 kDa, Figure 5B), and FGA (43–90 kDa, Figure 5C) in patients with early relapse compared with those with late relapse. The cutoff values were determined at expression levels that corresponded to the highest sensitivity and specificity, specifically 1.025, 0.900, and 1.050 for TPR, FGA, and JUP, respectively. Table 3 presents the correlations among EGFR-TKI relapse, other variables, and protein expression. Late relapse was strongly associated with elevated serum levels of FGA (*p* = 0.014) and TPR (*p* < 0.001). No significant associations were found between JUP expression and various clinicopathological variables, including sex, age, religion, smoking, drinking, or EGFR mutation.

### 3.8. Expression Levels of TPR, FGA, and JUP and Their Diagnostic Performance in the Serum of Patients with Lung ADC

**Figure 6:**
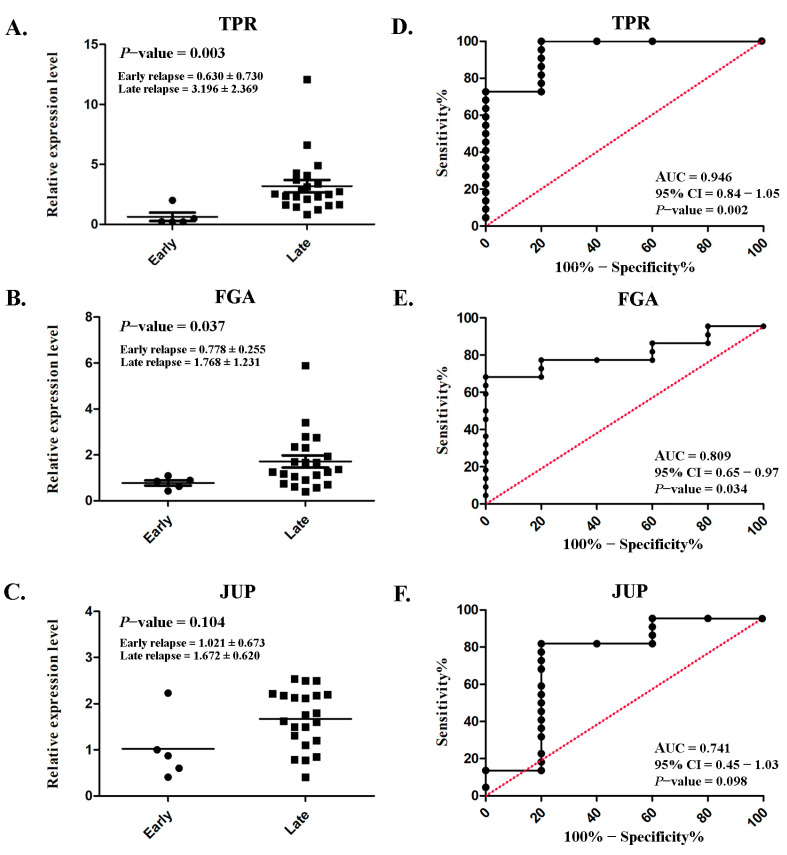
Serum expression levels and their diagnostic performance in lung ADC. Scatterplot illustrating the relative protein expression levels of TPR (**A**), FGA (**B**), and JUP (**C**) in patients with early relapse compared with those with late relapse. Analysis of the receiver operating characteristic curve for TPR (**D**), FGA (**E**), and JUP (**F**) to assess the diagnostic efficacy. Significant differences were determined using an independent *t*-test (*p* < 0.05).

Serum TPR and FGA expressions were markedly increased in patients with late relapse (3.196 ± 2.369 and 1.768 ± 1.231, respectively) compared with those with early relapse (0.630 ± 0.730 and 0.778 ± 0.255, respectively), with *p* = 0.003 and *p* = 0.037 (Figure 6A,B), respectively. However, no significant difference was observed in JUP (*p* = 0.104; Figure 6C). ROC analysis was conducted to assess diagnostic predictability. Satisfactory AUCs of 0.946 (95% CI: 0.84–1.05; *p* = 0.002; Figure 6D) and 0.809 (95% CI: 0.65–0.97; *p* = 0.034; Figure 6E) were achieved for TPR and FGA, respectively. Although a significant difference was not achieved, the AUC for JUP was fair (0.741; 95% CI: 0.45–1.03; *p* = 0.034; Figure 6F).

## 4. Discussion

Numerous biomarkers derived from liquid biopsies have been identified for cancer diagnosis and predictive treatment. Plasma and serum proteins, as a part of circulating liquid biopsies, serve as effectors of human biological activity. Thus, they are frequently studied in biomarker research and have several advantages, including higher stability, simplicity of detection, and minimal requirements for sample preservation and handling, over other circulating liquid biopsies [18]. In our present study, SWATH-MS was used for serum proteomic profiling. Only 14 proteins were differentially expressed between patients experiencing early and late relapse after EGFR-TKI treatment. This could be attributed to the complexity of the components, including lipids, salts, and other metabolites [19]. Moreover, serum, which is the blood component that remains after clotting, frequently contains fewer low-abundance proteins owing to factors such as degradation and posttranslational modifications during processing [20].

Among the 14 identified proteins, some genes or proteins that were previously identified in our transcriptomic or proteomic analyses of patients with lung cancer were associated with the response to chemotherapy. Proteomic analysis of patients treated with a doublet regimen of carboplatin and paclitaxel revealed elevated levels of IGHG3 in the serum of responders [16]. On transcriptomic analysis, SAA2 was upregulated in carboplatin- and paclitaxel-resistant cell lines, and on proteomic analysis, SAA2 was elevated in the serum of patients receiving the cisplatin and gemcitabine doublet regimen [17,21]. Patients receiving cisplatin and gemcitabine doublet regimens exhibited increased levels of FGA in both tissues and serum, as determined by transcriptomic and proteomic analyses [17]. This suggests that serum proteins are linked to diverse treatment responses and potentially can be employed as predictive markers for patients with lung ADC who receive either chemotherapy or EGFR-TKI treatment. Our online verification revealed an increase in three proteins (TPR, FGA, and JUP) that are associated with disease outcomes in tissues of patients with lung ADC. Following clinical confirmation in the serum, we found that increased expression of these proteins, especially TPR and FGA, was associated with acquired resistance and tumor recurrence. Furthermore, the diagnostic evaluation in the current study indicated that blood levels of TPA and FGA can help to effectively distinguish between patients with early relapse and those with late relapse on EGFR-TKI treatment.

Nucleoprotein TPR, also known as translocated promoter region protein or nuclear pore basket protein, is a 270 kD coiled-coil protein localized to the intranuclear filaments of the nuclear pore complex (NPC) that is involved in the nuclear export of macromolecules [22]. The TPR is one of the first NPC components to be associated with cancer. TPR is fused to the kinase domain of the MET oncogene, which leads to constitutive activation of MET [23]. Several studies have revealed that TPR can fuse with other kinase domains of oncogenes, including TRK in human papillary thyroid carcinomas [24], FGER1 in myeloproliferative syndrome [25], and ALK in lung adenocarcinoma [26]. One study suggested that TPR plays an oncogenic role by promoting the nuclear export of macromolecules, particularly several types of oncoproteins [27]. Chen et al. revealed that TPR is overexpressed in lung cancer cell lines as well as lung cancer tissues and is correlated with poor prognosis [28]. However, no recent evidence exists that supports the use of serum TPR as a potential biomarker of cancer. This study demonstrated that TPR serves not only as a significant tissue marker, but also as a blood-based biomarker for predicting response status.

FGA (or fibrinogen alpha chain) is a part of fibrinogen, which is made up of two pairs of three different polypeptide chains: alpha, beta, and gamma [29]. FGA protein peaks are highly expressed in gastric ADC [30], Stage I lung squamous cell carcinoma [31], and colon cancer [32], as demonstrated by several blood proteomics studies. Li et al. (2022) [33] found that FGA can be secreted from endometrial stromal cells into conditioned media. They also discovered that reduced FGA levels in conditioned media following FGA knockdown may suppress the angiogenic ability of endothelial cells through VEGFR2-FAK signaling [33]. In addition, a reduction in FGA level through CRISPR/Cas genome editing or short-hairpin RNA interference increases the cell proliferation, migration, invasion, and metastasis of lung ADC and gastric cancer cells via the integrin–AKT and FAK/ERK pathways [34,35]. Our previous studies revealed that FGA is present in both the tissue and serum of patients who are receiving a combination of cisplatin and gemcitabine. A high FGA level in the serum is associated with a high response to this regimen [17]. Our most recent findings support the findings of prior research, which indicated that FGA is involved in tumorigenesis and that elevated serum FGA levels can be used to predict both a response to doublet chemotherapy and a late relapse after a period of response to EGFR-TKI treatment. Nevertheless, the current investigation was restricted by the limited number of clinical samples, which may have affected the evaluation of the clinical utility of these proteins. Further clinical studies with larger sample sizes are required to confirm these findings.

## 5. Conclusions

This study demonstrated the presence of TPR and FGA in the sera of patients with lung ADC who were receiving EGFR-TKI therapy. TPR and FGA levels may serve as serum-based predictors of relapse after a period of response, and this suggests that patients with high TPR and FGA expression may have a prolonged response to EGFR-TKI therapy for lung ADC.

## Figures and Tables

**Table 1:** Clinicopathological characteristics of the participants (n = 27).

Variables	Category	Number (%)
Sex		
	Female	21 (77.8)
	Male	6 (22.2)
Age (years)		
	<60	6 (22.2)
	≥60	21 (77.8)
Religion		
	Buddhism	21 (77.8)
	Islam	6 (22.2)
Smoke		
	No	19 (68.0)
	Yes	8 (32.0)
Drink		
	No	25 (92.0)
	Yes	2 (8.0)
Recurrence		
	Early relapse	6 (22.2)
	Late relapse	21 (77.8)
Drug		
	Afatinib	2 (7.4)
	Erlotinib	20 (74.1)
	Gefitinib	5 (18.5)
EGFR mutation		
	Exon 18 G719X	1 (3.7)
	Exon 19 del	19 (70.4)
	Exon 21 L858R	7 (25.9)

**Table 2:** List of all differentially expressed serum proteins.

Gene Symbol	Protein Name	*p*-Value *	Log2 FC
TPR	Nucleoprotein TPR	0.001	3.00
IGHG3, IgG3	Immunoglobulin-heavy constant gamma 3	0.025	3.10
ABCF1	ATP-binding cassette sub-family F member 1	0.043	2.04
HSPBAP1	HSPB1-associated protein 1	0.010	2.03
FGA	Fibrinogen alpha chain	0.037	1.75
JUP	Junction plakoglobin	0.005	1.64
SAA2	Serum amyloid A-2 protein	0.011	1.54
VTN	Vitronectin	0.031	1.41
FINC, FN1	Fibronectin 1	0.009	1.30
APOE	Apolipoprotein E	0.018	1.19
AHSG	Alpha-2-HS-glycoprotein	0.005	1.10
KRT17	Keratin, type I cytoskeletal 17	0.018	1.05
KRT2	Keratin, type II cytoskeletal 2 epidermal	0.022	1.01
KRT10	Keratin, type I cytoskeletal 10	0.042	0.90

Abbreviations: Log2 FC, log2 fold-change; * significant differences identified using the independent *t*-test (*p* < 0.05).

**Table 3:** Association of clinicopathological variables and protein expression with recurrence in patients treated with EGFR-TKIs.

Variables		Treatment Response		*p*-Value
		Early	Late	
		Number (%)	Number (%)	
Sex				0.893
	Female	4 (80.0)	17 (77.3)	
	Male	1 (20.0)	5 (22.7)	
Age (years)				0.893
	<60	1 (20.0)	5 (22.7)	
	≥60	4 (80.0)	17 (77.3)	
Religion				0.893
	Buddhism	4 (80.0)	17 (77.3)	
	Islam	1 (20.0)	5 (22.7)	
Smoking				0.575
	No	3 (60.0)	16 (72.7)	
	Yes	2 (40.0)	6 (27.3)	
Drinking				0.234
	No	4 (80.0)	21 (95.5)	
	Yes	1 (20.0)	1 (0.5)	
EGFR mutation				0.532
	Exon 21 L858R	1 (20.0)	6 (27.3)	
	Exon 19 del	4 (80.0)	15 (68.2)	
	Exon 18 G719X	0 (0.0)	1 (4.5)	
FGA				0.014 *
	Low	4 (80.0)	5 (22.7)	
	High	1 (20.0)	17 (77.3)	
TPR				<0.001 *
	Low	4 (80.0)	1 (4.5)	
	High	1 (20.0)	21 (95.5)	
JUP				0.382
	Low	4 (80.0)	13 (59.1)	
	High	1 (20.0)	9 (40.9)	

* Comparison group showing significant differential expression with the chi-square test.

## Data Availability

Data may be provided with appropriate justification upon request.

## Abbreviations

The following abbreviations are used in this manuscript:

ADC	Adenocarcinoma
AUC	Area under the curve
CI	Confidence interval
CR	Complete response
DAVID	Database for Annotation, Visualization, and Integrated Discovery
DEPs	Differentially expressed proteins
DFS	Disease-free survival
DIA	Data-independent acquisition
EGFR	Epidermal growth factor receptor
EGFR-TKIs	Epidermal growth factor receptor tyrosine kinase inhibitors
FDR	False discovery rate
FGA	Fibrinogen alpha chain
GEPIA2	Gene Expression Profiling Interactive Analysis version 2
GO	Gene ontology
HR	Hazard ratios
HPA	Human Protein Atlas
IDA	Information-dependent acquisition
JUP	Junction plakoglobin
log2 FC	Log2 fold-change
MS	Mass spectrometry
NPC	Nuclear pore complex
NSCLC	Non-small-cell lung cancer
OS	Overall survival
PD	Progressive disease
PFS	Progression-free survival
PR	Partial response
RECIST	Response Evaluation Criteria in Solid Tumors
ROC	Receiver operating characteristic
SD	Stable disease
SDS-PAGE	Sodium dodecyl sulfate–polyacrylamide gel electrophoresis
SR plot	Science and Research online plot tool
SWATH-MS	Sequential Window Acquisition of all Theoretical fragment ion spectra mass spectrometry
TCGA	The Cancer Genome Atlas
TPR	Translocated promoter region
Tris-HCl	Tris hydrochloride
TOF	Time of flight
